# New Functions of Classical Compounds against Orofacial Inflammatory Lesions

**DOI:** 10.3390/medicines5040118

**Published:** 2018-11-01

**Authors:** Norifumi H. Moritani, Emilio Satoshi Hara, Satoshi Kubota

**Affiliations:** 1 Department of Oral and Maxillofacial Reconstructive Surgery, Okayama University Graduate School of Medicine, Dentistry and Pharmaceutical Sciences, Okayama 700-8558, Japan; hachi70@md.okayama-u.ac.jp; 2 Department of Biomaterials, Okayama University Graduate School of Medicine, Dentistry and Pharmaceutical Sciences, Okayama 700-8558, Japan; gmd421209@s.okayama-u.ac.jp; 3 Department of Biochemistry and Molecular Dentistry, Okayama University Graduate School of Medicine, Dentistry and Pharmaceutical Sciences, Okayama 700-8558, Japan

**Keywords:** stomatitis, recurrent aphthous stomatitis, oral lichen planus, CCN2, glucocorticoids, alkaloids

## Abstract

Anti-inflammatory agents have been widely used to ameliorate severe inflammatory symptoms of a number of diseases, and such therapeutics are particularly useful for diseases with intolerable pain without significant mortality. A typical example of this is a disease known as stomatitis; although stomatitis itself is not a life-threatening disease, it severely impairs the individual’s quality of life, and thus a standard therapeutic strategy for it has already been established. The topical application of a bioactive agent is quite easy, and a strong anti-inflammatory agent can be used without significant adverse effects. In contrast, natural products with relatively mild bioactivity are used for systemic intervention. However, new aspects of classical drugs used in these established therapeutic methods have recently been discovered, which is expanding the utility of these compounds to other oral diseases such as osteoarthritis of temporomandibular joints (TMJ-OA). In this review article, after summarizing the general concept and pathobiology of stomatitis, its established therapeutics are explained. Thereafter, recent advances in the research into related compounds, which is uncovering new biological functions of the agents used therein, are introduced. Indeed, regenerative therapeutics for TMJ-OA may be developed with the classical compounds currently being used.

## 1. Introduction

The oral cavity is a biological apparatus that enables the intake of food, drink, and air, thus enabling the intake of the most basic elements needed to support the development, growth, and maintenance of human bodies. Therefore, inflammatory lesions in the oral area are always deleterious to human health, impairing all of the biological activities of the cells constituting an individual. The most common inflammatory diseases in the oral region are those in, or around the teeth. Endodontic inflammation is where there is designated pulpitis, the acute form of which usually yields intolerable pain. However, owing to its poor regeneration potential, recovery of the affected pulp tissue can hardly be expected [1]. As such, a major intervention used to terminate pulpitis is a pulpectomy, which is the removal of the dental pulp under local anesthesia without anti-inflammatory treatment. In contrast, periodontitis is usually chronic and occasionally even asymptomatic. Needless to say, inflammatory responses of the host are critically involved in the etiology of periodontitis [2,3]. However, since periodontitis is based on the infection of a number of oral bacteria, the principal therapeutic methods against periodontitis have been the regulation of bacterial infection. For a better clinical outcome, the utilization of desiccants has recently been proposed [4], and acceleration of periodontal tissue regeneration by laser treatment has also been attempted [5,6]. In addition, the osseous drilling protocol has been regarded as critical for dental implants, rather than inflammation control [7]; therefore, anti-inflammatory molecules are not major agents in the treatment of these dental diseases.

Food intake can be more severely affected by stomatitis as any contact with the affected oral mucosa will cause great pain to the affected individual. Additionally, since it depends on mastication that is performed by the collaborative movement of temporomandibular joints (TMJ), osteoarthritis (OA) of the TMJ strongly restricts its quality and quantity. As will be mentioned in the next section, stomatitis is characterized by prominent inflammatory responses that have no apparent relationship with bacterial infection [8,9]. Notably, in contrast to the dental pulp, the regeneration potential of oral mucosa is quite high; as such, anti-inflammatory compounds have been widely used for the treatment of stomatitis to promptly redeem the individual’s food intake ability. In this article, starting from the review of stomatitis and related disorders, established therapeutics using classical compounds are summarized. Then, we report on the recent research topics that unveiled the possible utilities of a few such classical compounds in the treatment of the OA of TMJ for which no fundamental therapeutics have yet to be established. In particular, CCN family protein 2 (CCN2), a profibrotic protein involved in inflammation and tissue regeneration [10], has been found to mediate the novel biological effects therein. As such, we believe that the consideration of CCN2-mediated effects is critical in developing new therapeutic methods against inflammation.

## 2. Stomatitis and Oral Mucosal Diseases

**Figure 1:**
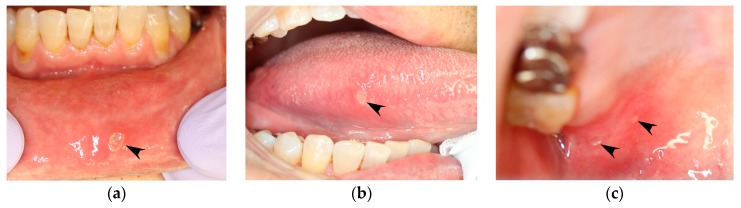
Aphthous stomatitis that occurs in a patient with recurrent aphthous stomatitis (RAS). Images are of the same patient and were taken on the same day. (**a**) Lower labial mucosa. (**b**) Right margin of the tongue. (**c**) Right hard palate mucosa (black arrowheads).

Stomatitis is a term commonly used to describe the inflammation of oral mucosa. The names of several stomatitis-related conditions are listed in Table 1. Among them, the most commonly recognized type of stomatitis is aphthous stomatitis. Aphthous stomatitis is where there are painful ulcers that are clearly defined as shallow, round, or oval, with a necrotic center covered by yellowish-tan pseudomembranes, and surrounded by an erythematous halo. An example of aphthous stomatitis is shown in Figure 1. The term “aphtha” refers to the clinical condition and is not the name of the disease; the appropriate disease name is recurrent aphthous stomatitis (RAS). As above-mentioned, stomatitis is a generic name for the inflammation of oral mucosa, and is also known as oral mucosal disease.

Oral mucosal diseases have numerous conditions such as those listed in Table 1. However, as we cannot present all such diseases, we have at least tried to classify oral mucosal diseases by their clinical manifestations, as shown in Table 2. Furthermore, the classification of the diseases listed in Table 2, according to etiology, is shown in Table 3. The oral mucosal diseases listed in Table 1, Table 2 and Table 3 pose a number of treatment challenges to clinicians such as: (1) few treatment opportunities being presented at clinics; (2) the cause being largely unknown; (3) symptomatic treatment is the mainstay of treatment, and not causal therapy, where a radical cure is difficult to achieve; and (4) that an efficacious therapy has not yet been established. It is probably recognized that the above-mentioned representative diseases are RAS and oral lichen planus (OLP).

### 2.1. Recurrent Aphthous Stomatitis (RAS)

RAS is also known as recurrent aphtha or recurrent aphthous ulcers. RAS occurs as single or multiple recurrences of aphtha occurring irregularly in the oral mucosa (Figure 1). The etiology of RAS is scientifically unclear, but we here show the conditions that are regarded as the etiology of RAS (Table 4). Since the etiology is unknown, its diagnosis is entirely based on history and clinical criteria, and no laboratory procedures exist to confirm the diagnosis.

Generally, the RAS ulcer is resolved spontaneously after a few days or up to 10 days, so the ulcer can be left untreated. However, the RAS ulcer may lead to difficulty in speaking, eating, and swallowing and, thus, may negatively affect the patient’s quality of life. As a result, topical therapies for the treatment of ulcers such as steroid ointment or mouthwash are often used. These therapies are directed at palliating symptoms and promoting the rapid healing of the RAS ulcer. However, there is no curative therapy to prevent the recurrence of ulcers, and all available treatment modalities only reduce the frequency or severity of the lesions. Although many causative factors have been proposed, the pathogenesis of RAS is still unknown, and a fundamental treatment for the disease has not been established. RAS is a cardinal symptom of Behçet’s disease, which is a systemic inflammatory disorder and is associated with a four-symptom complex of the oral mucosa, genitalia, eyes, and skin [8,9]. RAS occurs in Behçet’s disease in all cases and in the whole oral mucosa. Therefore, in the medical examination of patients with RAS, Behçet’s disease should be suspected, and a diagnosis to rule it out is necessary.

Behçet’s disease has been proposed to be caused by allergy, virus, and autoimmune-related mechanisms, but the exact etiology is still unknown. As for treatment, systemic therapies such as corticosteroid, immunosuppressant, anti-inflammatory, and anti-fibrinolysis medicines are used [11].

### 2.2. Oral Lichen Planus (OLP)

Oral lichen planus (OLP) is a relatively common diagnosis of oral lesions and has a prevalence of approximately 2% among oral mucosal diseases. OLP presents as reticular or plaque-like white lesions, which are chronic, passing inflammatory lesions with slight hyperkeratosis.

**Figure 2:**
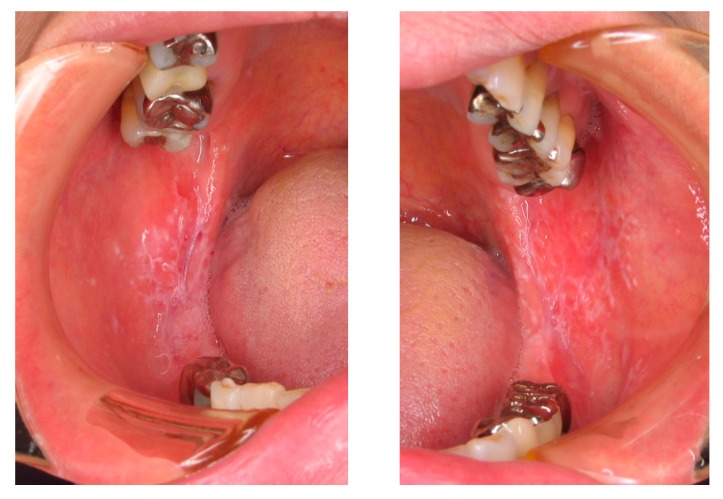
Oral lichen planus (OLP) in a patient, which occurred on both sides of the buccal mucosa with a white and papuloreticular lesion.

**Figure 3:**
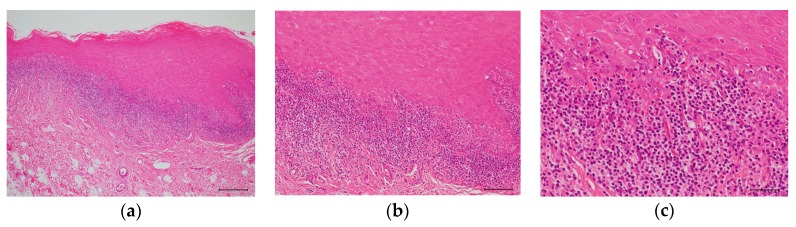
Hematoxylin and eosin (H&E) staining of an OLP tissue section. (**a**) Low power photomicrograph. Hyperkeratosis is presented on the surface of the epithelium and a band-like infiltrate of lymphocytes immediately subjacent to the epithelium. Scale bar: 100 µm. (**b**) Medium power photomicrograph. The rete ridge has a saw-toothed shape. Scale bar: 50 µm. (**c**) High power photomicrograph. Migration of lymphocytes into the lower epithelium is observed with liquefaction degeneration of the basal layer. The epithelial spinous cell layer directly contacts the infiltrating lymphocytes. Scale bar: 25 µm.

In typical cases, OLP occurs as a reticular white spot at the buccal mucosa (Figure 2). Occasionally, OLP occurs as erythematous lesions with erosion and ulcers. As these symptoms cause discomfort, contact pain, or both in affected subjects, OLP is an intractable disease that may impair the quality of life of patients. The clinical manifestation of OLP varies and presents as white spots that may be reticular, plaque-like, papular, linear, or circular and may occur together with atrophic erythematous, erosive, ulcerative, or rarely bullous-type lesions [12,13]. OLP is diagnosed based on clinical findings such as the previously mentioned pathognomonic gross appearance and the histopathological examination of lesional tissues by biopsy. Pathognomonic histopathological findings are shown in Figure 3. Predisposing factors are not clear yet, but some implicated etiological triggers or aggravating factors of OLP are shown in Table 5 [14,15,16].

## 3. Topical or Systemic Therapeutic Agents for RAS and OLP

**Figure 4:**
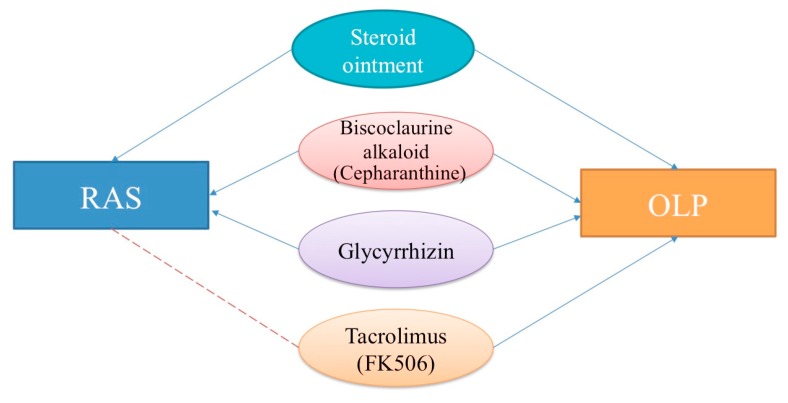
Therapeutic topical and systemic agents for recurrent aphthous stomatitis (RAS) and oral lichen planus (OLP). Blue lines with arrowheads: effective. Red dashed lines: probably effective.

As the same therapeutic agents are used for RAS and OLP, we mainly describe the medications used in this review (Figure 4). We consider that understanding the mechanism of action of the therapeutic agents for RAS and OLP might contribute to elucidating their etiology.

### 3.1. Glucocorticoids (Topical Use)

In Japan, Dexaltin oral ointment (proprietary name) 1 mg/g or Aphtasolon oral ointment (proprietary name) 0.1% are the major topical therapies used. Dexaltin and Aphtasolon 1.0 g both contain 1.0 mg dexamethasone (Dex), which is the active component. Dex is a corticosteroid glucocorticoid (GCs) and GCs are the most commonly used anti-inflammatory and immunosuppressive drugs. Topical GCs were introduced into medicine approximately 50 years ago. Corticosteroid strength is classified according to their effects in the vasoconstrictor assay and Dex showed the lowest potency in the ranking of a selected group of topical corticosteroid preparations [17]. Therefore, the incidence of associated adverse effects of topical applied steroid is low, and Dex-containing steroid ointments are often used as first choice medications for oral mucosal disease. The molecular mechanisms of the action of steroids include: (1) direct combination of the steroid–steroid receptor complex and DNA sequences called the steroid-responsive element; (2) interactions between the steroid–steroid receptor complex and other transcriptional factors, and (3) non-genomic pathways. The immunosuppressive activity results from the inhibition of neutrophil tissue infiltration, macrophages, eosinophils, and basophils. The anti-inflammatory activity results from both a decrease in the production of inflammatory cytokines and an increase in the production of anti-inflammatory cytokines [18,19]. After entry into the target cells, GCs bind to the GC receptor (GR), which then translocates into the nucleus to directly or indirectly regulate gene transcription. The ligand-activated GR binds as a homodimer to consensus sequences, termed as GC response elements, in the promoter region of GC-sensitive genes to induce the transcription (transactivation) of genes such as tyrosine aminotransferase (*TAT*). An indirect negative regulation of gene expression (transrepression) is achieved by GR-protein interaction. The ligand-activated receptor binds as a monomer to transcription factors such as nuclear factor (NF)-κb and activator protein-1 (AP-1) to inhibit the activity of many proinflammatory transcription factors. This transrepression is considered the key mechanism behind the anti-inflammatory activity of GCs.

#### 3.1.1. RAS

It is generally recognized that topical agents such as steroid ointments are the first treatment choice for RAS. Corticosteroids used in patients with RAS are intended to restrict the inflammatory process associated with the formation of aphthous stomatitis. Corticosteroids may act directly on T lymphocytes in the local environment and alter the response of effector cells to participants of immunopathogenic events. Neutrophils are also found in the surrounding tissue of the RAS ulcers at a marked concentration. The production of oxygen radicals by these neutrophils in RAS has been found to be similar to that in the controls [20].

#### 3.1.2. OLP

The topical use of steroids has been the standard treatment for OLP. In local legion sites of the OLP, oral steroid ointments suppress inflammatory changes and inhibit immune reactions. Therefore, oral steroid ointments are often used as first choice medications for OLP. However, the long-term, repeated application of steroid ointments may be associated with side effects such as candidiasis mainly, which requires attention [21].

### 3.2. Biscoclaurine Alkaloid (BA) and Cepharanthine (CEP)

Cepharanthine is an alkaloid extracted from the plant *Stephania cepharantha* Hayata, which is naturally found in the woods of southern Formosa, which is now in Taiwan. CEP is a member of a class of compounds known as biscoclaurine alkaloids (BAs). Alkaloids have long attracted the attention of pharmacologists and clinicians owing to their resemblance to polypeptides and their physiological action. It has been widely used in Japan to treat a number of acute and chronic diseases. CEP inhibits tumor necrosis factor (TNF)-α-mediated NF-κB stimulation, plasma membrane lipid peroxidation, and platelet aggregation, and suppresses cytokine production. CEP is recognized to exhibit reactive oxygen species (ROS)-scavenging properties and a protective effect against some of the responses mediated by pro-inflammatory cytokines including TNF-α, interleukin (IL)-1β, and IL-6 [22,23]. In addition, it has been reported that CEP has anti-allergic actions, stabilizes the biological membrane, augments the action of cortical hormones, and improves the peripheral circulation. In Japan, indications for CEP include radiation-induced leukopenia, alopecia areata, and alopecia pityrodes (Cepharanthine package insert, 2018). CEP has not demonstrated significant safety issues, and its side effects have been very rarely reported [22]. In addition, it is available for long-term treatment, and the treatment effect is persistent. Therefore, CEP is often used for OLP.

#### 3.2.1. RAS

RAS has been reported to apparently be related to the excessive generation of ROS including O_2_^−^ [24]. It has been reported that when CEP 3 g (30 mg per day as alkaloid extracted from the plant *S. cepharantha*) was administered daily in three divided doses after each meal for seven cases of RAS patients, the results showed a percent improvement of 71.4% and a response rate of 85.7% [25].

#### 3.2.2. OLP

It has been reported that the administration of BA effectively reduced the generation of O_2_^−^, one of the most potent reactive oxygen intermediates, and diminished the clinical symptoms of OLP [26]. When CEP was administered to patients with OLP, a significant suppression of O_2_^−^ generation by peripheral blood neutrophils was found as symptoms were improved (such as ulcers, redness, and contact pain of the oral mucosa). Therefore, O_2_^−^ excess generation of the OLP lesion was inhibited by the administration of CEP, and those symptoms were thought to be relieved. Moreover, it has been reported that the rate of plasma inflammatory cytokine production (such as TNF-α, IL-1β, IL-6, and granulocyte-colony stimulating factor (G-CSF)) was unchanged by CEP administration [24,27].

In addition, previous studies have reported improved microcirculation induced by the vasodilatory effect of CEP [28]. Furthermore, in previous studies, CEP 3 g (30 mg/day as the alkaloid extracted from the plant *S. cepharantha*) was administered daily in three divided doses after each meal to 12 patients with OLP [25]. The results indicated a percentage improvement and response rate of 75.0% for each [25]. These reports suggest that the suppression of O_2_^−^ generation by peripheral blood neutrophils may be closely related to the improvement of symptoms of RAS and OLP.

### 3.3. Glycyrrhizin (GL)

In Japan, glycyrrhizin (GL) is an established treatment for improving liver function in patients with viral hepatitis [29]. GL, which is a triterpenoid saponin isolated from the root of licorice (*Glycyrrhiza glabra*), is a compound consisting of a single glycyrrhetic acid (GA) molecule linked with two glucuronic acid molecules. GL exhibits multiple biological and pharmacological activities such as anti-inflammatory, anti-allergic, and antiviral [e.g., against herpes simplex virus (HSV), Varicella zoster, influenza, hepatitis C virus (HCV), and human immunodeficiency virus (HIV)] effects [30,31]. The mechanism of action of GL is not completely understood. However, a number of recent studies have indicated a number of distinct GL-binding functional proteins (GBFPs) as essential mediators, which are involved in the GL-induced anti-inflammatory effect. These proteins include arachidonate cascade-related enzymes [secretory phospholipase A_2_ (sPLA_2_), 5-lipoxygenase (5-Lox), and cyclooxygenase-2 (Cox-2), inducible nitric oxide synthase (iNOS), and the high mobility group box-1 protein (HMGB1)] [32]. One tablet of glycyron contains 25 mg glycyrrhizic acid as the main ingredient. In Japan, indication for GL includes stomatitis, the improvement of liver function abnormality in chronic hepatitis, eczema, dermatitis, alopecia areata, and strophulus infantum (Glycyron, package insert, 2018).

#### 3.3.1. RAS

Apparently, no previous study has reported the effect of GL on RAS. According to the Glycyron package insert (2018), when glycyron tablets are orally administered at a dose of nine tablets daily for 12 consecutive weeks, it is more than effective at 82.3%. Licorice, the name given to the roots and stolons of Glycyrrhiza species, has been used since ancient times as a traditional herbal remedy [33]. Some reports on the effect of licorice for controlling pain and reducing the healing time of aphthous ulcerations have been published [34,35,36]. However, these studies suggest that additional research is required to arrive at conclusions on the potential benefits of licorice in RAS [33]. Therefore, it is suggested that GL has a certain effect on RAS because of an effect that occurs in stomatitis (oral mucosal disease).

#### 3.3.2. OLP

Hasizume reported that when glycyron tablets were orally administered at a dose of six tablets daily for almost three consecutive months for OLP affecting the bilateral buccal mucosa with alcoholic chronic hepatitis, the symptoms were remitted [37]. In addition, Glycyrrhizin (GL) was used to treat chronic liver dysfunction in nine patients with OLP who were positive for HCV antibody and HCV RNA. GL was administered intravenously at a dose of 40 mL (0.2% solution) daily for four consecutive weeks, and the results showed that 66.7% of patients with OLP improved clinically [38].

### 3.4. Tacrolimus (FK506: Topical Use)

Tacrolimus, also called FK506, is a macrolide immunosuppressant produced by *Streptomyces tsukubaensis*, which has similar effects to those of cyclosporin A. It acts by inhibiting calcineurin, a ubiquitous calcium-dependent protein phosphatase that is responsible for immune responses [39]. Tacrolimus was formulated as an ointment for atopic dermatitis that is used commonly and seems effective as a second choice for treating OLP, which is refractory to other standard treatments [40]. Since the relationship between tacrolimus and cancer development has been reported in only a few cases, clinicians must be careful in selecting tacrolimus as a second-line treatment for OLP [41]. In RAS and OLP, immunopathy is regarded as one of the onset triggers. Therefore, if tacrolimus is effective for OLP, it is also expected that there is enough efficacy for RAS. However, tacrolimus is thought to have limited applicability for aphthous treatment as steroidal external preparations have a lower-risk than tacrolimus external preparations.

### 3.5. Desiccants (Topical Use)

Although not used in Japan, desiccants have been used for the treatment of RAS in the US and other countries. A typical desiccant cocktail contains sulfonic acid and other highly reactive agents, which destroys the attached cells [4]. Destruction of nerve endings results in the prompt relief of pain, which is followed by the regeneration of the destroyed tissue. Thus, the mechanism of action of desiccants can be regarded as the chemical removal of lesions, which is not related to the etiology of RAS. Nowadays, the utility of a desiccant in the treatment of periodontitis is being recognized [4].

## 4. Role of CCN Family 2 (CCN2) in Inflammation

Steroid hormone derivatives counteract inflammatory responses by inhibiting the action of proinflammatory transcription factors. In addition to this action, these molecules are known to activate the transcription of the *CCN2*, which encodes protein that is critical in wound healing. Therefore, while repressing the inflammatory response, glucocorticoids also promote the last stage of inflammation to reconstruct the damaged tissues.

The protein, CCN2 is a classical member of the CCN family, consisting of six members in mammals. CCN stands for the first letters of the initial names of its three founding members: cysteine-rich 61 (Cyr61/CCN1), connective tissue growth factor (CTGF/CCN2), and nephroblastoma-overexpressed (NOV/CCN3). CCN2 is composed of an insulin-like growth factor binding protein-like (I), von Willebrand factor type C repeat (V), thrompospondin 1 type 1 repeat (T), and C-terminal cystine-knot (C) modules. Multiple interactions via these modules with a variety of biomolecules in the microenvironment yield pleiotropic and context-dependent biological outcomes, which usually induce harmonized tissue development and regeneration [42,43,44]. Indeed, CCN2 is expressed at particular stages during the development of a variety of tissues and organs. After development and growth, CCN2 is transiently induced upon tissue injury and repair, and the tissue regeneration potential of CCN2 is also indicated [45,46]. CCN2 appears to play a pivotal role to proceed with inflammatory stages.

**Figure 5:**
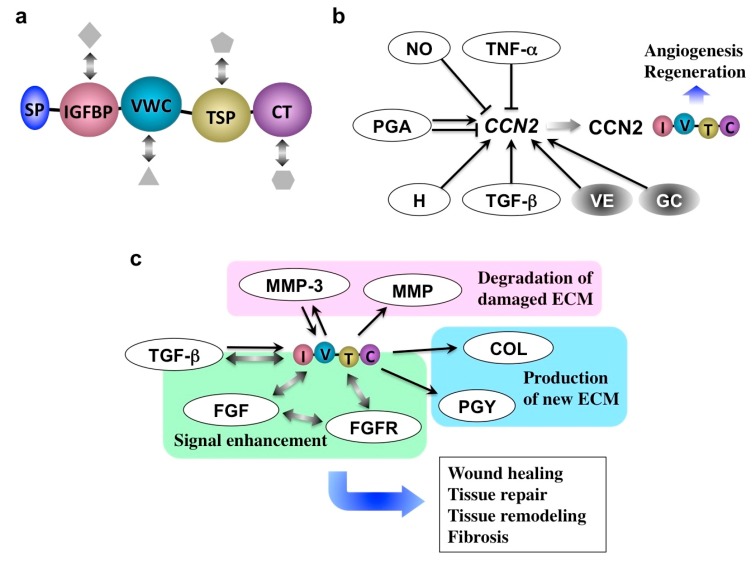
(**a**) Molecular structure of CCN2. Following the signal peptide for secretion (SP), insulin-like growth factor binding protein-like (IGFBP), von Willebrand factor type C repeat (VWC), thrombospondin type I repeat (TSP), and C-terminal cystine knot (CT) modules are connected in tandem. Interaction with multiple co-factors (objects in grey) that support the function of CCN2 is also illustrated. (**b**) CCN2 inducers and repressors. TNF-α, tumor necrosis factor alpha; NO, nitric oxide; PGA, prostaglandin; H, histamine; TGF-β, transforming growth factor beta; VE, vitamin E; GC, glucocorticoid. (**c**) Molecular action of CCN2. MMP, MMPs other than MMP-3; COL, collagen; PGY, proteoglycan; ECM, extracellular matrix; FGFR, fibroblast growth factor receptor; FGF, fibroblast growth factor. Arrows and T-bars indicate induction and repression, respectively. Bidirectional arrows denote direct molecular interactions.

As illustrated in Figure 5a, *CCN2* expression is differentially regulated by inflammatory mediators. Tumor necrosis factor-α (TNF-α) and nitric oxide repress *CCN2* expression in a variety of cells, whereas histamine contrarily induces it [10]. Moreover, CCN2 itself may enhance the gene expression of inflammatory cytokines in several types of cells. It is indicated that a processed CT module fragment of CCN2 is responsible for its inflammatory actions [47]. Such an apparently complex regulatory network around CCN2 during inflammation suggests that *CCN2* is precisely regulated in order to appear upon the initiation of the last stage of inflammation. Once the CCN2 protein is produced, this molecule starts reconstructing the damaged tissues under the direct interaction with other growth factors and their receptors. As a result, the production of matrix metalloproteinases (MMPs) as well as the extracellular matrix (ECM) components, is enhanced, which are then utilized for tissue reconstruction, as summarized in Figure 5b. Of note, MMP-3 was found to go back into the nuclei of producers to further enhance *CCN2* expression in chondrocytes, representing the collaborative action of CCN2 and MMP-3 [48,49].

After tissue repair, *CCN2* expression should be turned off immediately in order to avoid continuous tissue remodeling and excessive ECM production leading to fibrosis, a typical outcome of chronic inflammation [50,51,52,53]. Indeed, *CCN2* overexpression is commonly observed in fibrotic disorders in a variety of organs. Therefore, turning CCN2 production on and off are key for terminating acute and chronic inflammation, respectively. If we could turn on and off the CCN2 production by medication, we would thus be able to successfully control the inflammation and regeneration of affected tissues in a harmonized manner.

## 5. Novel Utility of Particular Glucocorticoid and Alkaloid in Orofacial Disorders

### 5.1. Fluocinolone Acetonide

**Figure 6:**
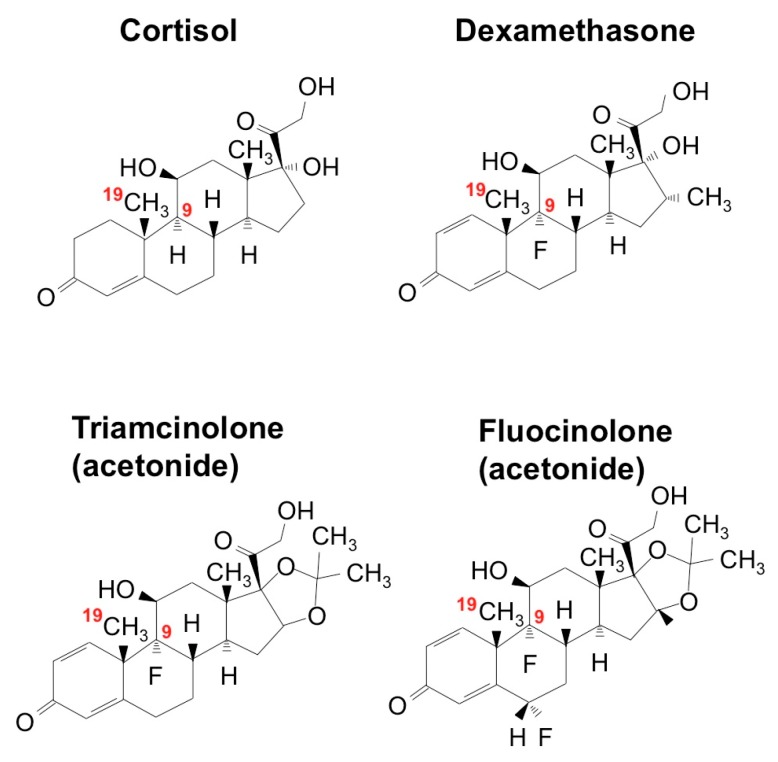
Chemical structures of glucocorticoids. The positions of C9 and C19 are indicated in red.

Due to their enhanced medical utility, most of the ointments used for stomatitis contain synthetic GCs with fluoride introduction at the position of C9 in the steroid nucleus (Figure 6). Dex is the most popular active ingredient, whereas triamcinolone acetonide (TA) is also employed. Another related compound, fluocinolone acetonide (FA) is commonly used in the field of dermatology in the form of an oil or paste, and its effectiveness and safety are also indicated in the treatment of stomatitis [54]. For a long time, the pharmacological effects of these synthetic glucocorticoids were believed to be basically the same. However, surprisingly, screening of the Food and Drug Administration (FDA) of the United States-approved small molecules rediscovered FA as a special molecule with novel biological potential to regenerate cartilage.

**Figure 7:**
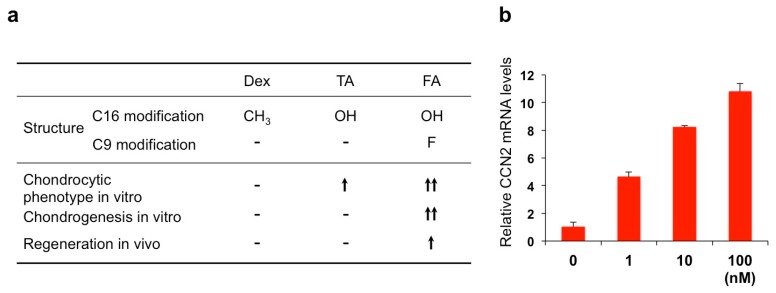
(**a**) Structural–functional relationship of three glucocorticoids. TA, triamcinolone acetonide; FA, fluocinolone acetonide. (**b**) Induction of CCN2 in ATDC5 chondrogenic cells by FA. ATDC5 cells in monolayer culture were treated with different concentrations (1–100 nM: horizontal axis) of FA for 24 h, and analyzed for expression levels of CCN2 mRNA.

**Figure 8:**
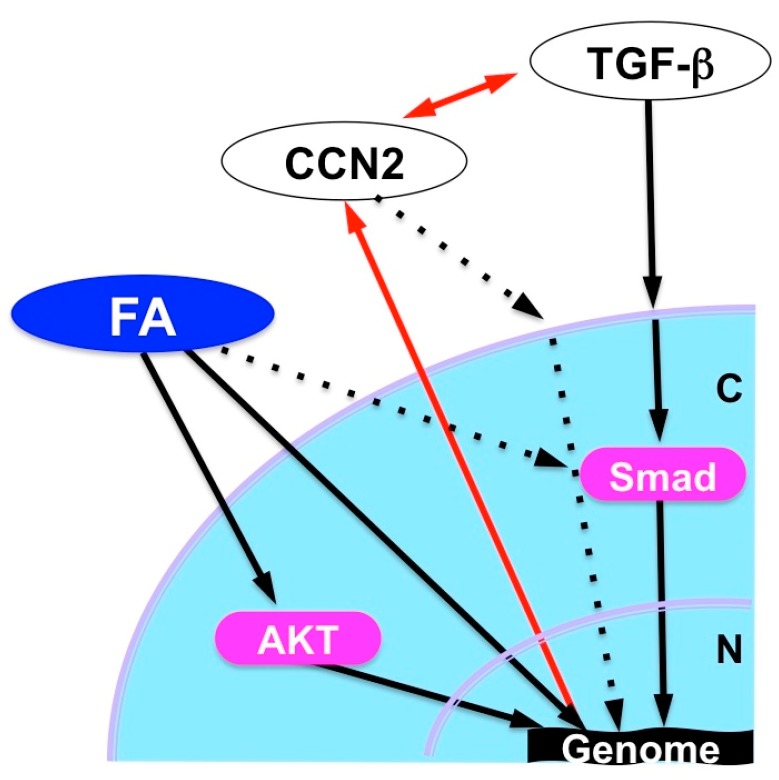
Possible cartilage regeneration mechanism by FA in collaboration with TGF-β. N, Nucleus; C, cytoplasm; FA, fluocinolone acetonide.

FA dramatically enhanced the chondrogenesis from mesenchymal stem cells in vitro when it was combined with transforming growth factor (TGF)-β3. Furthermore, analysis in vivo revealed the ability of FA to regenerate damaged articular cartilage in collaboration with TGF-β3 [55]. TGF-β3, as a member of the TGF-β superfamily, is known to enhance extracellular matrix deposition mainly through the canonical signaling pathway mediated by secondary messengers termed Smads. The observed effect of FA was shown to be mediated by the mammalian target of the rapamycin (mTOR)-AKT signaling pathway as well as by the interaction with the Smad pathway and GR activation. This collaboration is highly member-specific, since FA combined with BMP-2, another member of TGF-β superfamily, did not show such an effect. Similarly, articular cartilage regeneration in vivo was not effectively exerted by the combination of TGF-β3 and either TA or Dex. A structural comparison between these three compounds suggests a structural–functional relationship between corticosteroids and the extra functionality to regenerate cartilage in collaboration with TGF-β3 (Figure 7a) [55]. Obviously, the fluoride modification at C16 adds a structural property to enable the unusual collaboration of FA with TGF-β. It should also be noted that FA is able to strongly induce *CCN2* expression in ATDC5 chondrogenic cells, which may result in the modification of TGF-β signaling via direct molecular interaction between these molecules (Figure 7b). Since CCN2 itself regenerates articular cartilage, involvement of this protein in the outcome of FA-TGF-β3 collaboration is strongly suspected (Figure 8).

In the field of orofacial medicine, TMJ dysfunction based on osteoarthritis is one of the major complications that affects proper mastication. However, there are currently no therapeutics for regenerating damaged TMJ cartilage that has been established. However, FA has already been widely approved and is being used in clinics, meaning that its clinical application for recovering damaged TMJ cartilage is now expected, although particular care should be taken to avoid possible side effects as a glucocorticoid.

### 5.2. Harmine

**Figure 9:**
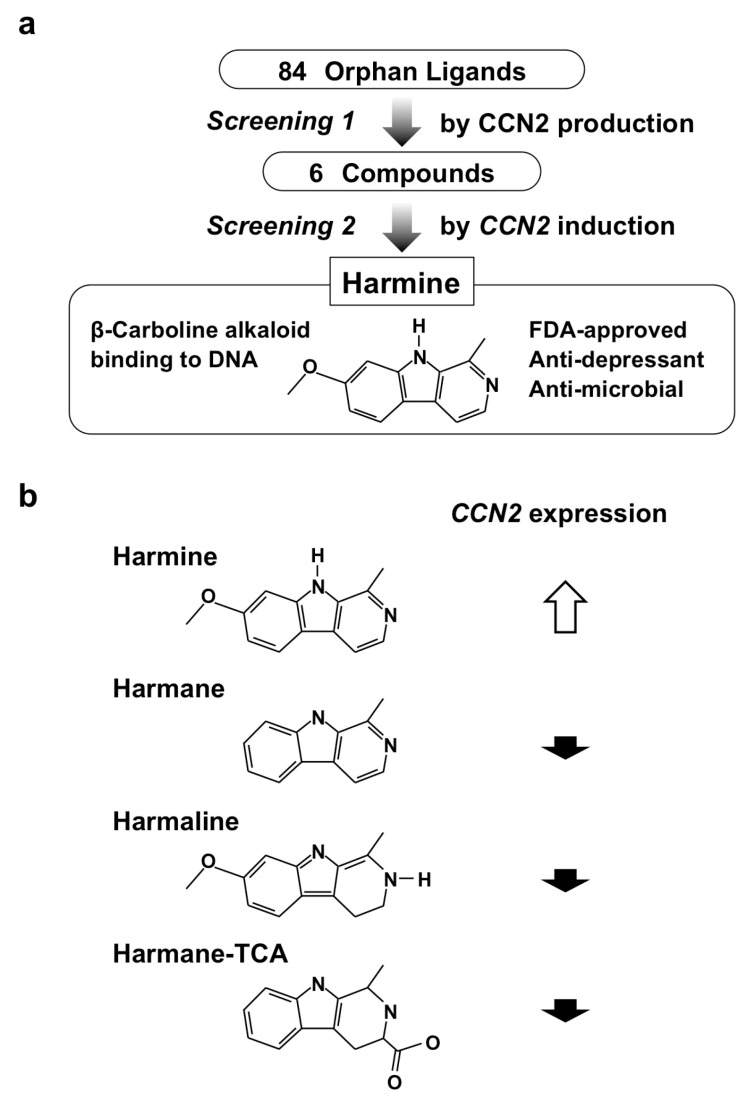
(**a**) Screening procedure employed for the re-discovery of harmine; (**b**) chemical structures and CCN2 inducing the activity of β-carboline alkaloids.

As a related attempt to study small molecules that enhance cartilage regeneration, Hara et al. also screened an orphan ligand library based on the ability to induce CCN2 expression. Among the 84 compounds screened, harmine, one of the β-carboline alkaloids, was found to be a potent inducer of CCN2 in the human chondrocytic cell line (Figure 9a). Harmine is a natural product that can be extracted from a plant named *Peganum harmala*, which has been used in folk medicine in a number of countries for thousands of years [56]. Later scientific studies showed an anti-depressant effect of this alkaloid via inhibiting monoamine oxidase activity and its vasorelaxant-like effect by blocking voltage-gated calcium channels. One should take these functions into account upon its clinical application to avoid possible adverse effects. The family of β-carboline alkaloids is composed of several members; however, harmine was confirmed to be the only one that could exert this novel effect to induce CCN2 expression (Figure 9b). Further investigation confirmed harmine’s ability to enhance chondrogenesis with the elevated gene expression of chondrocytic marker genes, as well as that of CCN2, in culture. Interestingly, enhanced production of SOX-9, which is a master transcription factor of chondrogenesis, was found to be stronger than that of CCN2. Therefore, harmine enhances chondrogenesis by inducing both CCN2 and SOX-9, which subsequently promotes the production of cartilaginous ECM components [57].

**Figure 10:**
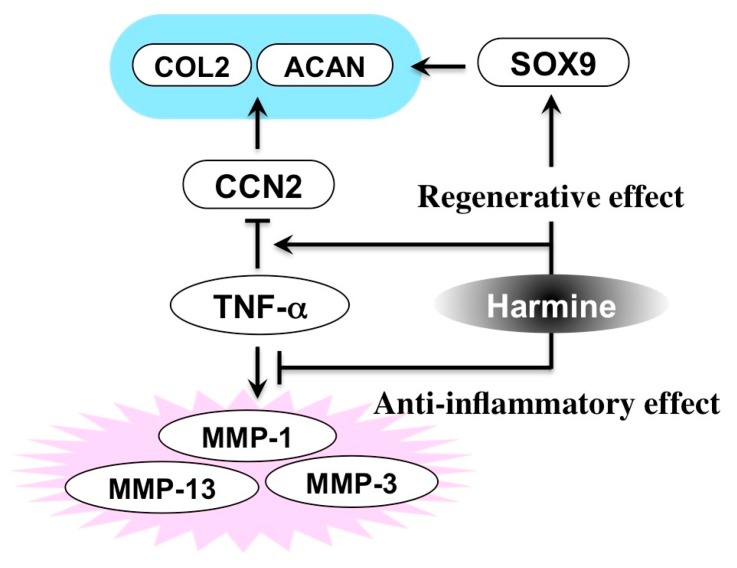
Molecular activity of harmine counteracting the inflammatory degradation of cartilage. COL2, type II collagen; ACAN, aggrecan; MMP, matrix metalloproteinase.

In addition to this novel discovery, the anti-inflammatory effect of this compound was surprisingly also indicated in the same study. Indeed, harmine was shown to protect chondrocytes from the catabolic effects conferred by an inflammatory cytokine, TNF-α (Figure 10) [57]. Therefore, therapeutic use of this compound in OA, including that of TMJ, could well be considered. Also, such a molecular function of this particular alkaloid, counteracting inflammatory response and enhancing regenerative activity, is reminiscent of that of glucocorticoids. Thus, harmine may be an effective agent for the treatment of stomatitis as well, which ought to be examined in future studies.

## 6. Conclusive Remarks

Inflammation in the oral region representative of stomatitis is not typically fatal but is highly painful, and thus strongly impairs an individual’s quality of life. Therefore, symptomatic, rather than fundamental treatment has been the major therapeutic strategy for these kinds of diseases. As such, glucocorticoids and alkaloids have commonly been employed to ameliorate local and systemic inflammatory responses for a long time, without significant advances in therapeutics and etiological research. However, recent research on these classical compounds is unveiling their utility in the regenerative therapy of damaged connective tissue, including intractable cartilage defects. These research outcomes also emphasize the close and delicate relationship between inflammation and tissue regeneration, in which CCN2 may be pivotally involved. Further investigation of other classical anti-inflammatory compounds may uncover unexpected molecular functions in those molecules as well, and the research outcome may be of help in exploring novel comprehensive therapeutics to terminate excessive inflammation and promote harmonized tissue regeneration in oral inflammatory lesions.

## Figures and Tables

**Table 1:** Stomatitis-related conditions.

Disease Names
aphthous stomatitis
recurrent aphthous stomatitis (RAS) (synonym: recurrent aphthous ulcer)
herpetic stomatitis
catarrhal stomatitis
drug-induced stomatitis
radiation-induced stomatitis
gangrenous stomatitis
nicotinic stomatitis
angular stomatitis (synonym: angular cheilitis)
denture-related stomatitis

**Table 2:** Oral mucosal disease; classification by clinical manifestations.

Major Symptoms	Diseases
bulla/vesicle	herpetic stomatitis, herpes zoster, pemphigus, pemphigoid, epidermolysis bullosa hereditaria
erythema, erosion	erythroplakia, drug-induced stomatitis, radiation-induced stomatitis, angular stomatitis, oral candidiasis, OLP
ulcer	RAS, Behçet’s disease, gangrenous stomatitis, denture-related stomatitis
white spot/patch	leukoplakia, OLP, nicotinic stomatitis
pigmentation	Peutz–Jeghers syndrome, melanin pigmentation
atrophic disease	glossitis with anemia (e.g., Hunter’s glossitis)

RAS, recurrent aphthous stomatitis; OLP, oral lichen planus.

**Table 3:** Oral mucosal disease; classification by etiology.

Etiology	Diseases
Congenital or developmental anomalies	epidermolysis bullosa hereditaria, Peutz–Jeghers syndrome
Physical or chemical cause	drug-induced stomatitis, radiation-induced stomatitis, nicotinic stomatitis, denture-related stomatitis
Bacterial infection	angular stomatitis, gangrenous stomatitis
Mycotic infection	oral candidiasis, angular stomatitis
Viral infection	herpetic stomatitis, herpes zoster
Allergic disease	drug-induced stomatitis, Quincke’s edema
Autoimmune disease	pemphigus, pemphigoid
Precancerous lesion	erythroplakia, leukoplakia
Unidentified or complex cause	RAS, Behçet’s disease, OLP

RAS, recurrent aphthous stomatitis; OLP, oral lichen planus.

**Table 4:** Proposed etiology of RAS.

Heredity/Genetic Factor
Local trauma such as sharp food and tooth-brushing
Adverse effect of drugs
Deficiency such as iron, zinc, vitamin B12, and folate
Smoking
Virus
Bacteria
Allergy
Hormonal change
Stress
Inflammatory digestive system disease
Immunological abnormality
Cardinal symptom of Behçet’s disease
Food hypersensitivity

**Table 5:** Proposed etiology of OLP.

Adverse Drug Reaction
Intraoral dental metal and filler
Intraoral cosmetics including preservatives, aromatic substances
Overwork and stress
Smoking
Hepatitis (hepatitis C in particular)
Oral candidiasis
Herpetic infection
Immunological abnormality
